# Rapid return to normal activities at a residential summer camp during the COVID-19 pandemic

**DOI:** 10.1007/s10389-021-01597-9

**Published:** 2021-09-02

**Authors:** A. Klunk, R. Holloway, A. Babaoff, E. B. Jelin

**Affiliations:** 1 Philadelphia College of Osteopathic Medicine, 4170 City Avenue, Philadelphia, PA 19131 USA; 2 Department of Bone Marrow Transplant and Immune Deficiency, Cincinnati Children’s Hospital Medical Center, 3333 Burnet Avenue, Cincinnati, OH 45229 USA; 3 University of Cincinnati College of Medicine, 3230 Eden Avenue, Cincinnati, OH 45267 USA; 4 Department of Emergency Medicine, Cincinnati Children’s Hospital Medical Center, 3333 Burnet Avenue, Cincinnati, OH 45229 USA; 5 Department of Surgery, Division of Pediatric Surgery, Johns Hopkins University, Johns Hopkins Charlotte R. Bloomberg Children’s Center 1800 Orleans Street, Baltimore, MD 21287 USA

**Keywords:** COVID-19, SARS-CoV-2, Residential camp, Serial testing, Infection prevention, Quarantine

## Abstract

**Aim:**

Infection prevention and control (IPC) within residential settings is a central focus of the coronavirus disease 2019 (COVID-19) pandemic. Youth residential summer camps are an excellent model for such environments and have thus far had mixed results. The aim of this report was to describe the successful implementation of a seven-week overnight summer camp with rapid return to normal activities from June to August 2020.

**Subjects and methods:**

This retrospective study included 427 individuals who traveled from 24 US states. All staff and campers were tested by serial nasopharyngeal PCR tests in the context of strict infection prevention and control (IPC) measures, including cohorts and masking. The entire camp population was isolated from non-camp personnel with special measures for food, supply, and mail delivery.

**Results:**

During the two-week staff session, one staff member tested positive for SARS-CoV-2, was isolated, and sent safely off premises. All other campers and staff had three negative PCR tests: 1–8 days before arrival, upon arrival, and 5–6 days after arrival. After these three negative tests, 6 days into camp, most IPCs, including masking, were successfully lifted and a normal camp experience was possible.

**Conclusions:**

These findings indicate that serial PCR-based testing and strict adherence to IPC measures among cohorts can allow for successful assumption of near normal group activities in a residential setting during the COVID-19 pandemic. This result at an overnight summer camp has broad implications for similar residential communities such as boarding schools, other youth education and development programs, as well as nursing homes and military installations.

**Supplementary Information:**

The online version contains supplementary material available at 10.1007/s10389-021-01597-9.

## Introduction

Individuals in residential environments such as university housing, nursing homes, long term care facilities, military installations, boarding schools, and other congregate settings are historically at increased risk for acquisition and transmission of infectious diseases compared to the average population (Dyal 2020). As such, preventive measures have often been employed to mitigate the spread of respiratory illnesses and other infectious diseases within these settings. The emergence of severe acute respiratory syndrome coronavirus 2 (SARS-CoV-2), the agent causing coronavirus disease 2019 (COVID-19), has dramatically increased the challenges facing residential communities. The widespread reach of this virus caused the World Health Organization to declare it a global pandemic on March 11, 2020 (Cucinotta and Vanelli 2020). As of November 16, 2020, there have been over 55 million cases worldwide, and over 11 million cases with over 250,000 deaths from COVID-19 in the United States [https://www.worldometers.info/coronavirus/#countries). Mitigation and prevention strategies have appropriately focused on social distancing and cessation of large group activities, which have led to decreased youth socialization and education; this decrease in activities has a negative impact on youth development as described in a recent statement from the American Academy of Pediatrics (American Academy of Pediatrics Pediatricians, 2020). In order to counter this negative impact and provide a safe opportunity for in-person youth interaction, we sought to use best epidemiologic and infectious disease evidence to design a safe youth residential overnight camp experience.

Our strategy was informed by the following understanding of SARS-CoV-2 epidemiology. The primary mechanism of virus transmission is through respiratory droplets from individuals infected with SARS-CoV-2. These droplets can also linger in the air as aerosols and infect individuals that come into contact with them (Gandhi et al. 2020; Meselson 2020; Morawska and Cao 2020; Machhi et al. 2020). Additional mechanisms of transmission include (1) direct bodily contact with an infected individual, (2) touching one’s eyes, nose, or mouth with contaminated hands (Li et al. 2020), and (3) contaminated surfaces (Rubens et al. 2020; Van Doremalen et al. 2020). In individuals who become infected with SARS-CoV-2, the median onset of symptoms occurs 5.1 days after infection (Lauer et al. 2020). Whereas knowing the incubation period is important for preventing spread, there is evidence that a proportion of the population have detectable viral loads and can transmit the virus prior to the development of symptoms and possibly without ever developing symptoms (Lee et al. 2020; Nishiura et al. 2020; Mizimoto et al., 2020; Kimball et al. 2020; Gudbjartsson et al. 2020; Zou et al. 2020). With the mechanisms of transmission, incubation period, and asymptomatic spread in mind, a strategy of serial testing, cohorting, and masking was developed for camp execution with planned enrollment of 126 staff and 301 campers. Serial testing was defined as sequential testing that traverses the incubation period of the virus. Assuming activity during this period is extremely low risk (e.g., effective quarantine), negative tests can establish true negatives at the end of the testing regime. Cohorting was defined as grouping the campers and counselors into pods of ten individuals or less that were as isolated as possible from one another to contain potential outbreaks to those pods (see methods for a more complete description).

Preliminary data has suggested that serial testing in the setting of quarantine can establish lack of infection or incubating infection in a population (Barocas et al. 2020). Additionally, a recent study conducted at Wyoming’s State Psychiatric Hospital suggests that with prompt identification and application of effective preventive measures, it is possible to minimize and eventually eliminate the spread of SARS-CoV-2 in the residential setting (Callaghan et al. 2020). Similar results were also obtained in skilled nursing facilities throughout Detroit, where repeated point prevalence surveys identified asymptomatic positive cases and allowed for implementation of infection control protocols, effectively reducing the rate of infection in patients and healthcare personnel from 35% to 18% (Sanchez et al. 2020). Extrapolating this preliminary data to the residential structure of youth summer camps, the question arose as to whether effective infection prevention and control (IPC) measures could minimize and eventually eliminate the spread of the SARS-CoV-2 virus at an overnight camp with a return of normal activities.

Highlighting the importance of this question, recent data from a residential summer camp in Georgia revealed a COVID-19 infection attack rate of 44% with as many as 76% of the individuals in attendance (346 campers and 251 staff) testing positive for SARS-CoV-2 (Szablewski 2020), resulting in forced early closure of this summer camp. Conversely, diligent use of IPC in combination with small cohorts and quarantining allowed four Maine residential summer camps to successfully execute camp sessions for 1022 campers and staff (Blaisdell et al. 2020). In these camps, functioning from June–August 2020, four asymptomatic individuals (0.4%) received positive SARS-CoV-2 tests prior to arrival; these individuals delayed arrival to camp after 10 days of isolation at home. Furthermore, one week after arrival at camp, 1006 attendees were tested via reverse transcription polymerase chain reaction (RT-PCR) testing; three asymptomatic individuals tested positive. These persons were isolated and cohorts underwent quarantining, and no secondary transmission occurred. This report, however, did not describe lifting of IPC protocols and assumption of normal camp activities; thus, the question arises as to whether serial testing and IPC protocols can allow for rapid return to normal camp activities. The purpose of this study is therefore to report the efficacy of serial testing, cohorting, and prevention measures on the rate of COVID-19 at a New Hampshire residential summer camp with specific emphasis on a return to normal camp programming. This report was conducted after exempt approval by the Johns Hopkins Institutional Review Board (IRB00261707).

## Methods

### Testing

SARS-CoV-2 infection was assessed using nasopharyngeal polymerase chain reaction (PCR) testing. Three, temporally spaced, nasopharyngeal PCR tests were performed on staff and campers in line with serial testing recommendations in New Hampshire State guidelines for reopening summer camps (Supplementary Fig. 1). Individuals were tested 1–8 days prior to their arrival at camp in their home location, upon their arrival at camp, and 5–6 days after their arrival. Additional testing was performed on any individual with suspicious symptoms throughout the duration of camp. All on site nasopharyngeal swab testing was performed by a single camp physician to ensure consistency. All tests were transported to regional hospitals or offices with CLIA certification for high complexity tests. PCR testing was performed on two separate platforms as cost and availability improved during the study period. The BioFire FilmArray Multiplex PCR system (bioMérieux, Marcy-L’Etoile, France) and the Panther Fusion System (Hologic, Marlborough, MA, USA) were used. Tests resulted within 12–24 h of specimen collection.

### Infection control

Infection control measures, including symptom and temperature screening (both prior to arrival and throughout the duration of the camp session), cohorting, hand washing, masking, social distancing, and disinfection, were implemented according to New Hampshire state guidelines for reopening summer camps (Supplementary Fig. 1). All staff arrived two weeks before the first day of camp for a 14-day quarantine period prior to camper arrival (all pre-arrival, on-arrival, and post-arrival tests were negative). In order to protect the camp community (in which all individuals had tested negative for SARS-CoV-2), strict criteria for entry onto camp premises was enforced. These included having only four drivers involved in transport of packages, mail, food, and supplies in and out of camp. These drivers were masked whenever in proximity to campers or staff, practiced social distancing, and were tested on a weekly basis for SARS-CoV-2 (all tests were negative).

### Cohorting

Staff cohorts were established prior to arrival at camp. Cohorts were based on mutual exposure prior to arrival and the geographical area from which individuals were traveling. All staff cohort groups contained ten individuals or less. Individuals remained in their cohort for all activities, meals, and sleeping. Cohort members were able to be unmasked around each other during meal and sleeping periods. Individuals were masked and practiced social distancing anytime they were in proximity to those outside their cohort. Staff remained in their cohort until after their third negative PCR test result 5–6 days into the pre-camp period, after which IPC measures were lifted and cohorts were able to interact without restriction.

Camper cohorts were established upon arrival to camp. Cohorts were based primarily on age, though age groups were further stratified by geographical area of travel and mutual exposure prior to arrival. All camper cohort groups contained ten individuals or fewer. Individuals remained in their cohorts for all activities, meals, and sleeping for the first 6 days of camp (third negative test). Cohort members were able to be unmasked around each other during meal and sleeping periods. Additionally, all cohorts remained in masks and socially distanced when they were near other groups until their third negative test. Campers were able to interact freely, unmasked after their third negative PCR test result (5–6 days into the camp session). The remaining four weeks of camp was conducted with nearly all IPC measures completely lifted (given no further positive COVID-19 cases appeared).

### Family engagement

Families agreed to take an active role in preparing their children for camp. All families monitored their campers’ temperatures and symptoms for the 14 days leading up to camp arrival and reported any abnormal temperatures/symptoms to camp healthcare personnel. In the weeks leading up to camp, up-to-date information and guidance was provided via several video calls accessible to all families. With assistance, families were able to obtain nasopharyngeal PCR testing for their camper(s) prior to arrival. Families also agreed to adhere to strict travel recommendations to camp per New Hampshire state guidelines.

## Results

### Demographics

**Fig. 1:**
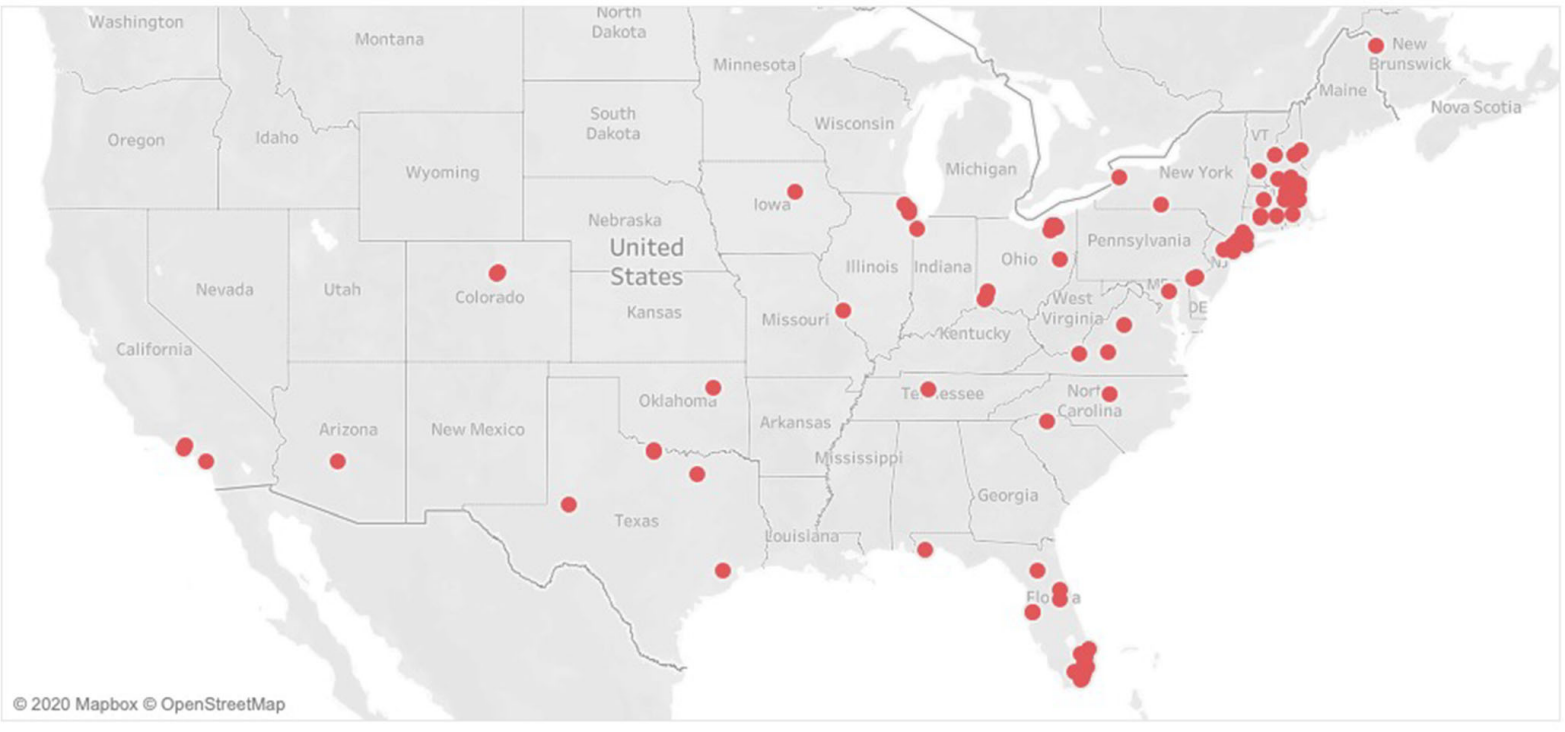
Map of staff departure locations to camp. Staff traveled from 24 states to reach Camp Robin Hood in Freedom, New Hampshire, with the majority traveling from New England

**Fig. 2:**
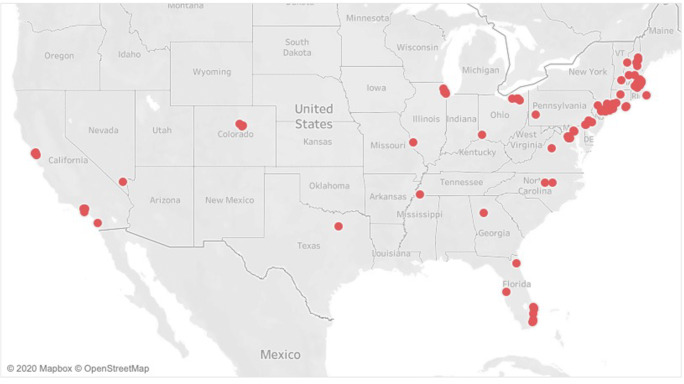
Map of campers’ departure locations to camp. Campers traveled from 18 states and Washington, D.C. to reach Camp Robin Hood

There were a total of 427 individuals at camp. There were 126 staff with the median age at 21 years (IQR 18–26 years). Fifty-two percent (66/126) of the staff was female. There were 301 campers with a median age of 12 years (IQR 10–14). Fifty-three percent of the campers were male (160/301). Staff traveled from 24 states to reach Camp Robin Hood in Freedom, New Hampshire. The majority of staff, however, arrived from the New England area (Fig. 1). Camper origins followed a similar pattern with arrivals from 18 states, and Washington, D.C. (Fig. 2).

### Staff testing and arrivals

Staff were required to have tested negative for SARS-CoV-2 by PCR within seven days prior to arrival. One staff member tested positive in pre-arrival testing. This staff member self-isolated at home and did not travel to camp. The remaining staff arrived two weeks prior to the arrival of campers at which point their “on arrival” PCR test was performed. One staff member (1/126, 0.8%) tested positive for SARS-CoV-2 on arrival. This staff member was isolated and sent off premises within 24 h. Those staff members who had come into contact with this positive individual were quarantined until their repeat SARS-CoV-2 tests resulted negative (see *SARS-CoV-2 Positive Procedures* below). The last round of staff testing was performed five days later at which point no staff tested positive for SARS-CoV-2.

### SARS-CoV-2 *Positive Procedures*

One staff member tested positive in the on-arrival testing with the result obtained within 24 h. This individual was immediately isolated from the remaining staff. Contact tracing was initiated at this time and individual interactions were placed into low, medium, or high risk categories. Individuals placed into medium and high risk categories were quarantined from the remaining staff until the next round of sequential testing. Individuals that were placed into the medium and high risk category were able to return to the staff cohort once their “day 5” SARS-CoV-2 PCR test resulted negative.

### Camper testing and arrivals

**Fig. 3:**
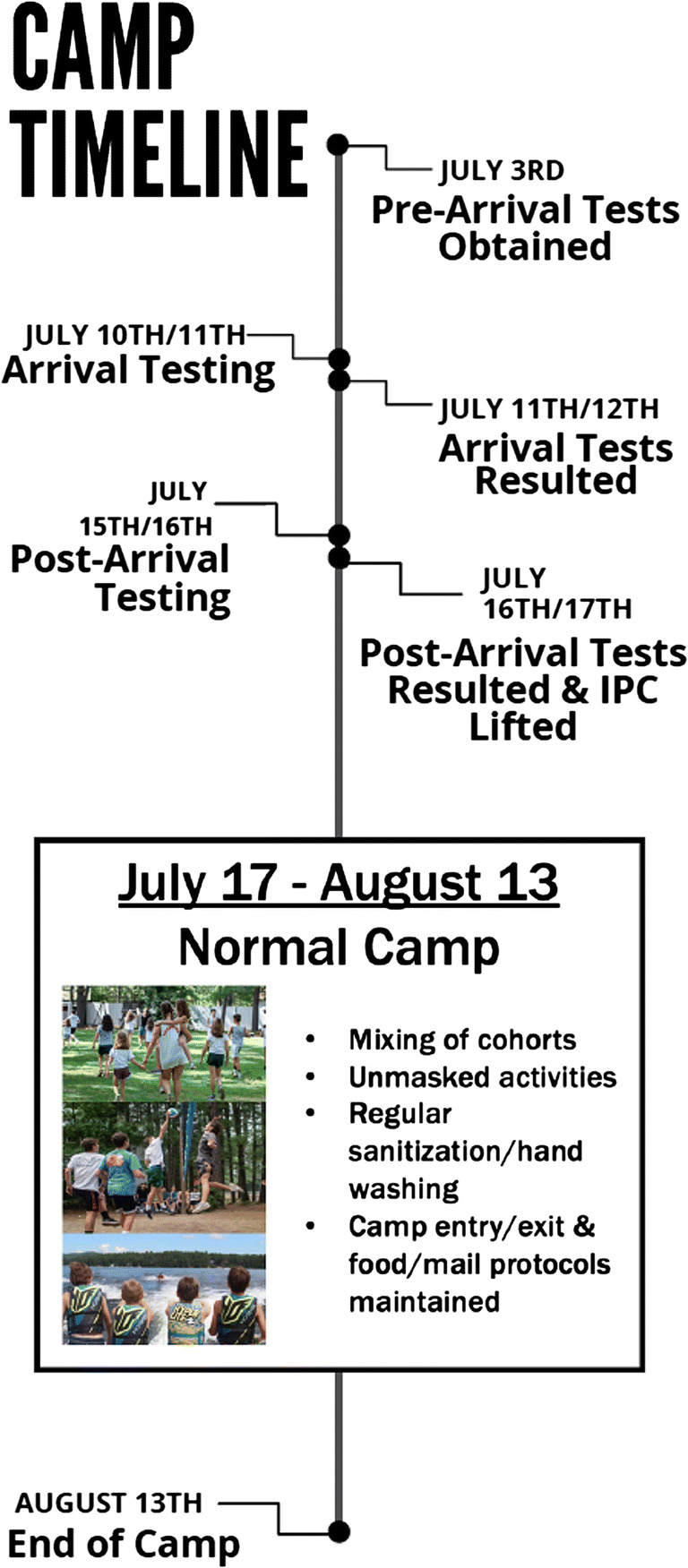
Timeline for camp testing, lifting of IPC protocols, and resumption of normal camp activities

Campers were required to have tested negative for SARS-CoV-2 by PCR 1–8 days prior to arrival. All campers tested negative in the pre-arrival testing. The same sequential testing was performed on campers as for staff. All campers tested negative for their “on arrival” testing and their “day 5” camp PCR test (Fig. 3).

### Testing surveillance

During the seven-week camp program four individuals were tested for COVID-19 suspicion apart from routine surveillance. One individual was a high-risk contact of the staff member who tested positive on arrival at camp. Two individuals were tested due to unexplained gastrointestinal symptoms (abdominal pain, vomiting, or diarrhea) and one camper was tested due to unexplained fever. These individuals experienced symptoms at various time points during camp. All tests were negative.

### Lifting of IPC protocols

Outside of one asymptomatic staff member who tested positive on arrival, all other staff on-arrival and post-arrival SARS-CoV-2 tests resulted negative. Additionally, all post-arrival camper SARS-CoV-2 tests obtained 5–6 days into the camp session resulted negative. For each camper, this was the third SARS-CoV-2 test performed, and all had resulted negative. Thus, through close communication with the state of New Hampshire, camp physicians obtained confirmation that IPC protocols, including cohorting and masking, could be lifted. Within six days of camper arrival, cohorts were freely mixed and masks were removed (Fig. 3). However, frequent disinfection and hand washing was still strictly enforced, and temperature and symptom screening was performed daily. All subsequent SARS-CoV-2 testing in patients with concerning symptoms throughout the remainder of camp resulted negative.

## Discussion

In this retrospective report of a seven-week New Hampshire residential summer camp during the COVID-19 pandemic, we detailed the efficacy of IPC measures on the rate of SARS-CoV-2 infection and transmission, as well as reported the ability to return to essentially normal activities after six days. A total of 427 individuals were in attendance at camp, including 126 staff members and 301 campers. Staff members were required to arrive at camp two weeks prior to campers to complete an initial isolation period, totaling seven weeks at camp. New Hampshire state guidelines were used as a minimum framework for IPC measures. Preventive measures in the first week of camp included serial nasopharyngeal PCR testing, mandatory masking, cohorting, social distancing, avoiding indoor or close-contact situations, travel restrictions, daily symptom/temperature tracking, and contact tracing. After the third negative test for all individuals on-site approximately six days into the camper session, most IPC measures were readily lifted (cohort expansion and mask removal) and a nearly normal camp experience was safely achieved. This outcome shows that with appropriate IPC and testing, it is safe and feasible to conduct a residential summer camp that draws from a wide swath of the United States, including areas with high positivity rates, without SARS CoV-2 transmission. This finding is particularly relevant as it exists in stark contrast to a recent Georgia overnight summer camp which was forced to close due to 260 confirmed positive cases (Szablewski 2020). Furthermore, our findings fall in line with recent data from four Maine residential summer camps that successfully implemented camp with proper multifaceted IPC measures and risk stratification (Blaisdell et al., 2020). However, the findings in this study deviate from the Maine camps in that IPC measures were lifted (cohort integration and removal of masking) six days into the camp term, and the remainder of camp was conducted in near normalcy without any positive SARS-CoV-2 cases (Fig. 3).

In order to create this COVID-free experience, effective education and frequent communication with families, as well as stringent family/camper adherence to social distancing and masking, was essential. Prior to camp, virtual visits were conducted with all families in which emphasis was placed on following CDC guidelines, selectively quarantining at home, and monitoring symptoms/temperatures in preparation for camp arrival. These virtual visits also increased trust between campers, their families, camp administration, and healthcare personnel, allowing families to feel comfortable sending their children to camp. Camper and family compliance was critical to decreasing SARS-CoV-2 exposures, monitoring all symptoms/temperatures, and thus increasing the likelihood of a COVID-free camp experience. In addition, serial testing with rapid results (within 24 h of test collection) in the context of strict cohorting of staff and campers contributed critically to the camp’s success. These measures limited exposure of individuals to small units to prevent wide transmission from an asymptomatic SARS-CoV-2 positive individual. This mechanism functioned well for the staff member who tested positive upon arrival and only had two high risk contacts. This experience is concordant with previous data showing that frequent testing allows for timely identification of positive SARS-CoV-2 cases (Barocas et al. 2020; Callaghan et al. 2020; Sanchez et al. 2020), demonstrating that carefully planned interval testing among cohorts can allow for successful resumption of near normal group activities.

This finding at a summer camp has broad implications for similar residential communities. The United Nations estimated that over 90% of children have been out of school or confined to online learning since the start of the pandemic (Child 2020). A study conducted in China by Ma et al. (2021) found through a well developed survey that 56.4% of respondents did not feel that online education was effective for gaining knowledge. The survey also demonstrated an increase in depressive symptoms and PTSD for students since the onset of the pandemic. Of the students surveyed, those experiencing the most profound effects were middle school and boarding school students. Implementation of the model used at this summer camp could help return students to boarding schools quickly and safely. This model could be applied to facilities such as colleges, nursing homes, and military installations. A challenge, however, to application of this model is the degree of isolation that could be achieved within a camp that may not be possible in other settings (see limitations). Lastly, this study provides more evidence that asymptomatic surveillance testing may be able to take center stage in broader, less controlled environments to certify the safety of individuals taking part in potentially risky but important economic activities.

### Limitations

This report has several limitations. First, the degree of IPC protocol adherence was not directly measured. Furthermore, Camp Robin Hood has its own campus that could be effectively isolated from “outside” contact. That is, once on campgrounds, staff and campers had little to no contact with persons outside of camp, minimizing risk of outside exposure. We acknowledge that near complete isolation from the rest of society is difficult to obtain in many settings. These factors must be taken into consideration when evaluating whether conducting a residential program would be feasible. Additionally, prevention and infection control measures were implemented by the camp, which might not be feasible in all residential situations depending on the organization’s financial status and available workforce.

### Future directions

This report shows that execution of a residential summer camp with appropriate infection surveillance and control measures is feasible and safe during the COVID-19 pandemic. Despite certain limitations, the results of this report may be applicable to the safe functioning of other residential settings, including universities, boarding schools, nursing homes, long term care facilities and military installations. It may even have applications in settings such as professional sports teams and other non-residential, yet closely cohorted groups of people. Future studies of SARS-CoV-2 infection in educational settings should explore the impact of effective serial testing, social distancing, and cohorting among students to allow them to return to the classroom.

## Supplementary information

Supplementary Fig. 1 Guidelines for re-opening overnight summer camps in New Hampshire (PDF 1855 kb)
