# Supplementary material for: Rapid return to normal activities at a residential summer camp during the COVID-19 pandemic
Source: Z Gesundh Wiss. 2021 Sep 2;30(11):2657–63. doi: 10.1007/s10389-021-01597-9 (PMC8412395; doi:10.1007/s10389-021-01597-9)
Supplement: Supplementary file 1 — Guidelines for re-opening overnight summer camps in New Hampshire (PDF 1855 kb) [file 10389_2021_1597_MOESM1_ESM.pdf]

Compare Results

Old File:		New File:
**guidance-overnight-camps_1.pdf**	versus	**guidance-overnight-camps_1 (2).pdf**
**17 pages (1.36 MB)**		**18 pages (1.39 MB)**
6/5/20, 3:03:08 PM		6/16/20, 11:14:17 AM

Total Changes

44

Text only comparison

Content

8 Replacements

22 Insertions

14 Deletions

Styling and Annotations

0 Styling

0 Annotations

Go to First Change (page 2)

Safer at Home
OVERNIGHT CAMPS

COVID–19 REOPENING GUIDANCE
GOVERNOR'S ECONOMIC REOPENING TASKFORCE

Safeguarding Guidance:

The State of New Hampshire recommends the Resident/Overnight Camp industry establish measures to protect consumers and employees. The following guidance is built upon recommendations from the Universal Guidelines for All New Hampshire Employers and Employees, the CDC and additional resources created by Environmental Health & Engineering, an independent multidisciplinary consulting company in public health, workplace safety, research and data analytics which has been retained by ACA and YMCA-USA to provide educational resources and guidance for camps to function as effective public health partners in the current COVID-19 environment. This guidance was developed through the formation of an independent expert panel, which included members of the American Academy of Pediatrics, the Association of Camp Nursing and the Harvard School of Public Health, in dialogue with the CDC.

- Review and follow the updated NH Universal Guideline
- Review and follow the NH day camp guidance.
- Review and follow CDC considerations for youth and summer camps.

Considerations:

1. Many Residential Camps, with acceptable modifications, can quarantine, functioning as a 'single family home' and 'shelter-in-place' together for the duration of the camp session regardless of camp size.
2. Camp age children and staff represent the lowest-risk segment of the population for COVID-19 complications and health care utilization.
3. Travel and group gathering restrictions should be considered in the context of residential summer camps ability to **self-isolate**.
    a. Camps are committed to transportation options that limit exposure of out-of-state campers and staff to their local NH communities including:
        i. Direct-to-camp/direct-to-home parental/guardian transportation
        ii. Only staff and campers that are able to drive to the camp and be picked-up by car or other privately chartered ground transportation.
4. Overnight camp programs will keep campers on the camp premise for the duration of camp program, with direct-to-camp/direct-to-home transportation.
5. Visitors (see also Visitors, Parents & Field Trips) will not be allowed on camp property during periods of group quarantine. Visitors will be limited to essential service providers for the duration of the camp session. Essential visitors must don facial coverings when in camp.

Preparation Requirements:

1. Work with medical directors and other medical staff (e.g. camp nurses) to develop a COVID-19 Camp Plan (i.e., a COVID-19 specific Communicable Disease Plan) which specifies a plan and process for:
2. Systematically performing temperature and symptom screening on all staff, adults, teenagers, and children on the camp.
3. Testing all staff, volunteers, and campers for COVID-19 as discussed below.
4. Develop training, secure supplies and capacity to collecting, handle and shipping nasopharyngeal or other allowable/recommended swabs for COVID-19 testing.
5. Routine and frequent cleaning and disinfection.
6. Isolation of symptomatic people and quarantine of anybody exposed to a person who is undergoing COVID-19 testing or has confirmed COVID-19 (see below for more information on developing an isolation and quarantine plan).
7. Other guidance outlined in this document, the NH Universal Guidelines, and CDC considerations for youth and summer camps.
8. Strongly encourage camps to identify a local New Hampshire medical director for the camp who is an M.D., D.O., or APRN (note: a medical "consultant" is not adequate to meet this requirement). The medical director should be involved in developing, reviewing, and approving the COVID-19 Camp Plan, and be able to help manage any staff or campers who either develop symptoms consistent with COVID-19 (i.e., suspected to have COVID-19), or are confirmed to have COVID-19. This individual should also, under their medical or nursing license, order testing for COVID-19 (discussed below in section on testing requirements).
9. Train your medical staff on use of appropriate COVID-19 personal protective equipment (PPE), which involves at a minimum wearing a medical gown, gloves, surgical face mask, and eye protection (face shield or goggles) when evaluating a person with COVID-19 symptoms. If there is an aerosol generating procedure (e.g., nebulizer treatment), then an N95 mask or higher level respirator is needed when evaluating and treating a patient.
10. All out of state arriving staff and volunteers must arrive at the camp at least 14 days before the official start of camp. They must not leave the camp grounds during this time or go into the surrounding community for recreation, groceries, laundry, etc. Laundry facilities must be supplied on camp grounds. During this 14-day pre-camp session, staff and volunteers should be undergoing temperature and symptom monitoring as outlined above in addition to the COVID-19 testing requirements below.
11. Any campers coming from out-of-state must quarantine in NH for at least 14 days before camp, or attest to home quarantine in their home state in the 14 days before start of camp session and not go out in public places or interact with others outside of their household during their home quarantine or in the period of time between home quarantine and arriving at camp (e.g., en-route to the camp).

12. Staff, volunteers, and campers must receive pre-arrival screening by asking about symptoms of COVID-19 or risk factors for exposure outlined in the NH Universal Guidelines. Pre-arrival screening must be conducted in the 7-days before arrive and ideally should utilize a self-screening tool that is answered and available to the camp BEFORE arrival at the camp.
13. Camps will not allow any person with symptoms of COVID-19 or an identified risk for COVID-19 exposure into the camp.
14. Health screening will be also conducted upon arrival at the camp.
15. Camps must identify "safety officers" from their staff whose job it is to monitor and improve compliance with these guidelines, and social distancing, hand hygiene, cloth face covering use, and cleaning and disinfection policies.
16. Develop a drop-off and pick-up process which staggers arrival/departure of children and parents/guardians so that children and parents/guardians from different groups do not interact. Attempt to also stagger drop-off and pick-up times to avoid congregating of parents and children within a facility.
17. Alter all camp processes to incorporate social distancing and avoid close contact between staff, volunteers, and campers.

Employee & Consumer (camper) Protection

1. **Health Screening/Surveillance**

   a. **Pre-Arrival & Arrival:** Camp administrators may use examples of screening methods in CDC's supplemental Guidance for Child Care Programs that Remain Open as a guide for screening children and CDC's General Business FAQs for screening staff.

      1. Pre-arrival Screening of Campers and Staff- Recommend use of a pre-arrival screening by having campers and staff complete a self-screening tool during a 7-10 day period prior to their arrival at camp and provide it to the camp during check-in procedures. In addition, staff must meet Universal Guidelines for All New Hampshire Employers and Employees for arrival screening.

      2. Camps will not admit staff who are symptomatic for COVID-19. Implement health screenings in accordance with any applicable privacy laws or regulations. Confidentiality should be maintained.

      3. Consider including specific questions regarding COVID-19 symptoms and temperature monitoring in the health screening process.

      4. Health screening will be done upon arrival of staff and campers and throughout the camp experience at the discretion of the

camp medical staff and in accordance with camp's Communicable Disease Plan.

b. **Daily**- Screen campers and staff daily for symptoms of COVID-19 throughout the camp program. Staff must meet Universal Guidelines for All New Hampshire Employers and Employees for daily health screening.
c. **Worker Health**- Upon arrival, screen staff for symptoms of COVID-19 and exposures to positive COVID-19. Staff will arrive at camp and remain at camp for the duration of their quarantine. Consider minimizing or eliminating staff travel off camp facilities for the duration of the summer. Local NH staff may need to travel back and forth to camp each day but will wear PPE appropriate to their job function while at camp when social distancing cannot be maintained. Local staff will follow safety protocols to minimize the potential for COVID spread within the camp community.
d. **End of Program-** Daily surveillance will continue through the end of camp.

2. Temperature and Symptom Screening, and Testing Requirements:

a) All staff, volunteers, campers, and anybody else on camp premise must have their temperature taken and be screened/questioned for the presence of any COVID-19 symptoms on arrival to camp and daily thereafter during their stay at camp. All persons should be questioned about the presence of the following symptoms:
   - Fever (including subjective fever) or chills
   - Cough
   - Shortness of breath or difficulty breathing
   - Severe Fatigue
   - Muscle or body aches
   - New loss of taste or smell
   - Sore throat
   - Congestion or runny nose
   - Nausea or vomiting
   - Diarrhea

b) Log and keep a record of all people that have their temperature taken (record temperatures) and are screened for symptoms daily.

c) Any person (staff, volunteer, or camper) who develops any of the above symptoms must be tested for COVID-19 using an appropriately collected nasopharyngeal swab, which is the best type of specimen (i.e. highest yield) for diagnosing COVID-19. Specimen collection should be performed by medical staff on the camp premise to avoid needing to

transport a person off camp property and potentially exposing others (e.g. person driving the car).

d) Camp medical directors should be the ordering clinician for all COVID-19 specimen collection and testing conducted through the camp.

e) All staff and volunteers at the camp must be tested for COVID-19 as follows:

   1.) Nasopharyngeal swabs should be collected to test directly for the presence of the SARS-CoV-2 novel coronavirus that causes COVID-19 using a polymerase chain reaction (PCR) based test within 7 days before arrival at the camp. This specimen collection and testing should occur through a person's normal healthcare provider or health system. Results must be back before a person can arrive at the camp. Nobody may arrive to the camp who tests positive.

   2.) Upon arrival to the camp, and during the first day of the person's 14-day pre-camp period at the camp (before campers arrive), all staff must be tested a second time using a test to directly detect the presence of the SARS-CoV-2 novel coronavirus that causes COVID-19 using a polymerase chain reaction (PCR) based test. Ideally this should involve a second nasopharyngeal swab because of the higher test sensitivity (ability to pick up infection); however, if specimen collection using a nasopharyngeal swab is not possible to obtain on camp premise for large scale surveillance testing of staff and campers, the camp can utilize a nasal mid-turbinate or anterior nares (nostril) swab, being sure to follow appropriate instructions on specimen collection, as outlined in CDC recommendations (note: symptomatic individuals should still be tested using a nasopharyngeal swab whenever possible). Mouth swabs or saliva samples are not recommended due to lower ability to detect infection. Similarly non-PCR based tests, such as antigen tests (e.g., Quidel, Coris BioConcept) are not appropriate tests at this time for testing of all staff and campers. Specimen collection must occur at the camp and utilize the camp's medical staff or contracted providers, and process developed and outlined in the COVID-19 Camp Plan. Specimens must be appropriately collected, handled, and shipped to the contracting laboratory performing the expedited testing. Medical staff performing the testing must utilize appropriate PPE, and change necessary PPE and perform hand hygiene between persons per existing CDC and NH DHHS guidance, and as outlined in the COVID-19 Camp Plan developed with camp's medical director.

3.) All staff should be tested a third time at day 7 during their 14-day on-camp quarantine period using the same process outlined above. These test results must be back before the official beginning of camp and arrival of campers.

- Records must be kept of all testing and test results. Results must be kept confidential in compliance with state and federal HIPAA regulations.

- Anybody testing positive should be immediately isolated.

- **If there is a confirmed case of COVID-19, the camp must contact the Bureau of Infection Disease Control (BIDC) immediately at 603-271-4496 (available 24/7).**

f) All campers must be tested for COVID-19 as follows:

a) Nasopharyngeal swabs should be collected to test directly for the presence of the SARS-CoV-2 novel coronavirus that causes COVID-19 using a polymerase chain reaction (PCR) based test within 7 days before arrival at the camp. This specimen collection and testing should occur through a person's normal healthcare provider or health system. Results must be back before a person can arrive at the camp. Nobody may arrive to the camp who tests positive.

b) Upon arrival to the camp, and during the first day of the camper's arrival, all campers must be tested a second time using a test to directly detect the presence of the SARS-CoV-2 novel coronavirus that causes COVID-19 using a polymerase chain reaction (PCR) based test. Ideally this should involve a second nasopharyngeal swab because of the higher test sensitivity (ability to pick up infection); however, if specimen collection using a nasopharyngeal swab is not possible to obtain on camp premise for large scale surveillance testing of staff and campers, the camp can utilize a nasal mid-turbinate or anterior nares (nostril) swab, being sure to follow appropriate instructions on specimen collection, as outlined in CDC recommendations (note: symptomatic individuals should still be tested using a nasopharyngeal swab whenever possible). Mouth swabs or saliva samples are not recommended due to lower ability to detect infection. Similarly non-PCR based tests, such as antigen tests (e.g., Quidel, Coris BioConcept) are not appropriate tests at this time for testing of all staff and campers. Specimen collection must occur at the camp and utilize the camp's medical staff or contracted provider, and process developed and outlined in the COVID-19 Camp Plan. Ideally this testing would be incorporated into the camp check-in process. Specimens must be appropriately collected, handled, and shipped to the contracting laboratory performing the expedited testing. Medical staff performing the

testing must utilize appropriate PPE, and change necessary PPE and perform hand hygiene between persons per existing CDC and NH DHHS guidance, and as outlined in the COVID-19 Camp Plan developed with camp's medical director.

c) All campers that are staying for longer than 2 weeks should also have a third PCR-based test during their second week of camp (ideally day 7 similar to staff process above).

- Records must be kept of all testing and test results. Results must be kept confidential in compliance with state and federal HIPAA regulations.

- Camper's will not be allowed on camp if parents/guardians do not give consent for testing as outlined in this document, including testing on an ad-hoc basis if camper becomes symptomatic (see below).

- In addition to the routine testing required above, any person on the camp who develops symptoms of COVID-19 (even mild singular symptoms outlined above) identified on daily temperature and symptom screening must be tested for COVID-19 utilizing the same process and test outlined above.

- Anybody who is symptomatic and undergoing testing should be removed from other people and group interaction (isolated) until test results return as discussed below.

- Anybody who tests positive must be immediately isolated.

- **If there is a confirmed case of COVID-19, the camp must contact the Bureau of Infection Disease Control (BIDC) immediately at 603-271-4496 (available 24/7).**

3. COVID-19 and COVID-19-Like Illness Management

a. Health Centers:

1. Monitor Health Center logs to identify illness patterns.
2. Consider adjusting medication administration processes in the Health Center to promote social distancing.
3. Consider implementing a strategy for triaging individuals in the Health Center that promotes social distancing.
4. Train Health Center staff to follow camp communicable disease strategies: don/doff PPE, steps in Communicable Disease Plan (CDP), health screening activities, etc.

5. Create a system for camps to communicate with public health officials, nearby healthcare facilities, families, and other stakeholders.
6. Create a communication system for staff and families to self-report symptoms and notification of exposures.

b. When a camper or staff is identified with potential COVID-19 symptoms, this person will be isolated and quarantined from camp activities until COVID-19 status can be determined.

1. Camps must develop locations for isolation and quarantine of staff, volunteers, or campers if needed.
2. Isolation is for people who have tested positive for COVID-19, or who have symptoms of COVID-19. Isolation refers to the act of staying away from other people and out of public spaces to avoid spreading infection.
3. Quarantine is for people who have been in close contact to someone diagnosed with or suspected of having COVID-19. Quarantine refers to the act of staying away from other people and out of public spaces to avoid spreading infection in the event the exposed person develops infection (could be either symptomatic or asymptomatic infection).
4. Isolation locations should include a separate room with a separate bathroom in a location separate from others, ideally with a private entrance.
5. Any staff, volunteer, or camper who develops symptoms of COVID-19 should be isolated pending test results and may not participate in any camp activities, go to the dining hall, or be present in other public places.
6. If a person is confirmed to have COVID-19, they must leave camp immediately and be picked up by family and brought back home by private transportation. The family and close household contacts will then need to quarantine due to exposure to a person with COVID-19. People with confirmed COVID-19 shall not, under any circumstances, be allowed on public transportation. If a person is unable to drive themselves home (for staff and volunteers), or be picked up by family, they must continue to be kept in isolation on camp premise.
7. Symptomatic persons or those with confirmed COVID-19 should undergo enhanced medical monitor to ensure stability in health. Camps should have a detailed plan for how to transport a symptomatic person to the local hospital in the event the person needs medical attention. Transportation should involve a mechanism for avoiding exposing individuals to COVID-19 (e.g., consider utilizing local emergency medical services).
8. Any person requiring quarantine due to COVID-19 exposure should leave the camp and can either drive themselves home (for staff and

volunteers), or be picked up by family. People under quarantine shall not be allowed on public transportation. If a person is unable to leave the camp or be picked up immediately, they should undergo quarantine on camp premise.

9. Quarantine locations should ideally involve a private room with a private bathroom in a location separate from others, ideally with a private entrance. Whole groups/cabins who have all been exposed to a person with COVID-19 could theoretically quarantine together (with their own private bathroom), but if additional cases are identified the group would be under a rolling quarantine extending the need to quarantine for longer than 14 days (quarantine is for 14 days after last known exposure).
10. Any staff, volunteer, or camper who is exposed to COVID-19 and must be quarantined may not participate in any camp activities, go to the dining hall, or be present in other public places.
11. People under quarantine should undergo enhanced monitoring as outlined in the NH DHHS self-quarantine guide.
12. Food, laundry, and other essential services and needs must continue to be provided to people under isolation and quarantine, but the person(s) must not come into close or direct contact with other people.
13. Camp healthcare providers should use Standard and Transmission-Based Precautions when caring for sick people. See: What Healthcare Personnel Should Know About Caring for Patients with Confirmed or Possible COVID-19 Infection.
14. In the event of a confirmed case by viral PCR testing, notify State and local health officials as required, staff, and families in accord with the CDP while maintaining confidentiality as required by the Americans with Disabilities Act (ADA). Perform contact tracing to ascertain information and identification of close contacts.
15. Clear and close off recent areas used by an ill camper/staff and do not use before cleaning and disinfection. Ensure safe and correct application of disinfectants by staff and keep disinfectant products away from children. Arrange for a deep cleaning of the camper's residential area and/or the staff's workspace.
16. Adjust camper and staff policies to reflect the need for a COVID-19-suspected or COVID-19-positive individual to be immediately isolated from the larger camp community.
17. If camper or staff are confirmed positive for COVID-19, continue isolation according to your CDP, test when possible. Advise staff members to not return to camp until they have met CDC criteria to discontinue home isolation.

4. Prevention

a. **Physical Distancing, Group Size & Cohorting:** Residential camps will comply with physical distancing and group size guidelines as described below. Additionally, campers may be divided into small cohorts with same counselors. Cohorts should ideally remain together throughout the program and limit interaction with other groups as much as possible.

   1. Camp administrators should ensure campers and staff are separated into groups that remain consistent over the camp program. Consider programs that function by bunk and dining/activity cohorts that are groups of bunks. CDC guidance in Phase 2 states that "Social settings of more than 50 people, where appropriate distancing may not be practical, should be avoided unless precautionary measures are observed." Mixing between cohort groups of 50 should be discouraged.
   2. Space seating indoors and outdoors at least 6 feet apart between camper groups (for example, separate bunks/cabins groups by 6 feet apart in a recreational hall).
   3. Consider limiting large gatherings, events, and extracurricular activities to those that can maintain social distancing and support proper hand hygiene. Outdoor fields can be used for large gatherings with at least 6 feet maintained between camp groups of up to 50 persons. Manage communal use spaces, shared facilities, and playgrounds to avoid large gatherings; stagger times of use and disinfect in between use.
   4. When staff are within their cohorts they are not required to wear cloth face coverings

b. **Sleeping Arrangements:**

   1. Campers must be divided into small cohorts with the same counselors for the duration of the camp session. These counselor/camper groups must not exceed 10 total people (e.g., 2 counsellors and 8 campers). Cohorts must remain together throughout the camp session and limit interaction with other groups.
   2. For camps that have sleeping areas that accommodate larger groups, camps should divide the sleeping area so that no more than 10 total people are sleeping in the same shared space.
   3. These groups should maintain a consistent sleeping arrangement (i.e., no moving between cabins or bunks). Beds must be arranged so that campers and staff sleep head-to-toe. Ideally beds should be spaced at least 6 feet apart. If beds are unable to be spaced 6 feet apart due to limited space, beds should be spaced so that one

camper's/staff's head is more than 6 feet from an adjacent camper's/staff's head space.

4. Windows in sleeping areas/cabins must remain open as much as possible to increase ventilation.
5. Appropriate counsellor/camper ratios should be maintained.

c. **Meals:**

1. Stagger meal times.
2. Meal and snack time should occur outside whenever possible under tents, shelters or pavilions, if necessary. No congregating in large groups to eat meals and snacks.
3. Staff and campers should eat with their own group and maintain at least 6 feet of distance between camper groups when seated and eating; no sharing of food, drink, or utensils.
4. If meals must be provided in a dining hall/room, stagger meal times, arrange tables to ensure that there is at least six feet of space between groups, and clean tables between lunch shifts.
5. Serve individually plated or boxed meals.
6. Avoid buffet lines.
7. Avoid family style meals. If meals must be served family-style, campers should be served by camp counsellors. Campers may not touch serving utensils or serve themselves.
8. Hand hygiene must be performed by everybody before and after meal times, and also immediately before serving food.
9. Develop a process for clearing and cleaning & disinfecting tables one group at a time that avoids interaction between staff and campers.
10. Campers and staff need to bring their own water bottles. No shared water jugs.

d. **Limit Sharing**

1. Attempt to keep each camper's belongings separated from others' and in individually labeled containers, cubbies, or areas. Avoid sharing clothing, personal care products, and belongings between campers and between staff.
2. Ensure adequate supplies to minimize sharing of high-touch materials assigned to a single camper (art supplies, sports equipment, etc.) or limit use of supplies and equipment by one group of children at a time and clean and disinfect between use.
3. Have pre-packaged boxes or individual bags of snacks to avoid sharing by campers and staff.

e. **Programmatic Considerations**

1. Train all staff
    - Train all staff in the above safety actions. Consider conducting the training to ensure that social distancing and healthy

hygiene practices are maintained.

- Training of staff in infection prevention measures for this summer is essential. Camp administrators should train staff on the Communicable Disease Plan (CDP) and clarify staff's essential role in the plan.

2. Activity Considerations: Camps will alter programmatic activities to reflect current recommendations for physical distancing & group size. When physical distancing is not possible outside of their camp cohort, face coverings should be used.
3. Wilderness Activities: Camps with overnight wilderness trips will ensure that trip groups are consistent with their camp cohort. Trips will be in small groups of no more than 10. Travel trips will be done with the cohort and face coverings will be encouraged in the vehicle. Trips will minimize the number of campers in each tent.
4. Camp administrators should restrict arrival to camp and departure from camp to the greatest extent possible. Camps should consider having campers and staff with direct camper contact (for example, bunk counselors, activity and program leaders) remain on campgrounds for the duration of the camp session with exceptions to outside medical or other essential visits. Campers leaving camp and returning should wear cloth masks when social distancing is not possible and should avoid visiting public areas such as restaurants and retail settings. Upon return to camp, all campers and staff must go through pre-arrival screening and conduct hygiene practices as described within this document.
5. Arrangements should be made for retaining staff with direct camper contact on premises on days off.
6. Visitors & Parents & Field Trips:
   - Restrict nonessential visitors, entertainers, volunteers, and activities involving outside groups.
   - Field trips, socials and intercamp games to public gathering and recreational places should be avoided. It may be possible to permit small groups to day travel to nearby recreational areas where interfacing with the external community is not expected. For example, taking campers for equestrian sessions, transporting cyclists to go mountain biking or campers traveling offsite for a canoe trip.
7. Dining schedules should be altered or staggered to adhere to the maximum group gathering guidelines. Alternative to buffet style serving, camps could consider serving meals 'family style" to minimize movement and exposures in the dining facility. Counselors will serve their bunk campers.
8. Parent Visiting Weekend: Visitors, including parents, will be minimized to every extent possible, including the elimination of

parent visiting days. If a visit by a parent or other visitor becomes essential to camp, face coverings will be used when social distancing is not possible and interactions with the larger camp community will be minimized.

9. Inter-camp Games & Socials: Inter-camp athletics and social activities between camps will not be allowed.

f. **Hygiene**

a. Promotion of Health Practices:

- Teach and reinforce washing hands and covering coughs and sneezes among children and staff.
- Teach use of cloth face coverings among staff working outside their cabin or programming cohorts when physical distancing is not possible. Staff should be frequently reminded not to touch the face covering and to wash their hands frequently. Information should be provided to all staff on proper use, removal, and disposal or washing of cloth face coverings.
- Staff that travel off-camp every day and at the end of their shift should wear cloth face coverings in camp when unable to maintain a 6-foot distance from others.
- Have adequate supplies to support healthy hygiene behaviors, including soap and water, hand sanitizer with at least 60 percent alcohol for staff and older children who can safely use hand sanitizer, tissues, and no-touch trash cans.
- Post signs on how to stop the spread of COVID-19, properly wash hands, promote everyday protective measures, and properly wear a face covering (staff).
- Provide educational materials in advance to parents and guardians for sharing with children prior to camp and reinforce awareness at staff and camper orientation and periodically thereafter for all throughout the camp experience.

b. Face Coverings: When physical distancing is not possible outside of the cabin and program cohorts, use of face coverings are encouraged. Additionally, dining staff should wear face coverings when serving food. It is not advisable for campers to sleep with face coverings.

c. Health Center: Health staff will wear face coverings when physical distancing cannot be maintained. PPE for health staff will be consistent with CDC guidelines for its use in suspected communicable disease including the consideration of N-95 respirators, procedural masks, gowns, and eye coverings.

d. Facilities & Vendor Deliveries: Facilities staff and vendors will wear face coverings when physical distancing cannot be maintained and for the former when in camper living areas.

5. **Face Coverings**:
    a. All camp staff and volunteers are encouraged to wear reusable/washable cloth face coverings over their nose and mouth as much as possible when social distancing is not possible to help prevent the spread of COVID-19.
    b. Provide training on cloth face coverings based on CDC guidance for Use of Cloth Face Coverings.
    c. Review the NH DHHS information about using cloth face coverings.
    d. People wearing face coverings must not touch their eyes, nose, mouth, or face, or adjust their face covering without first sanitizing hands. After touching face or adjusting face covering, hands must be sanitized.
    e. All adults dropping children off at day camp should be asked to wear a cloth face covering over their nose and mouth when at the day camp facility or public spaces where other individuals are present.
    f. Any staff or volunteers who need to leave the camp premise for essential purposes must wear a cloth face covering when with other people or out in public settings when social distancing is not possible.
    g. The NH Department of Health and Human Services does not recommend young children routinely wear face masks or face coverings
6. **High Risk Populations:** Vulnerable or high-risk populations require special consideration at day and residential camps.
    a. Camp directors should advise staff members and campers' parents to consult with their primary care providers to determine if camp is a reasonably safe option for them.
    b. Families of campers with high risk individuals residing in their homes must consider COVID-19 exposure risks if they send their child to camp and determine if it is safe.
    c. Camp directors should follow CDC and White House *Opening Up America Again* plan that specifically state that special high-risk and vulnerable populations should continue to shelter-in-place through Phases 1 and 2. Specifically, camp directors should consider if the following populations should seek employment at summer camp facilities with consult from their primary care provider:
        i. People 65 or older
        ii. People who live in a nursing home or long-term care facility
        iii. People of all ages with underlying medical conditions, particularly if not well controlled including:
            1. People with chronic lung disease or moderate to severe asthma
            2. People who have serious heart conditions

3. People who are immunocompromised: Many conditions can cause a person to be immunocompromised, including cancer treatment, smoking, bone marrow or organ transplantation, immune deficiencies, poorly controlled HIV or AIDS, and prolonged use of corticosteroids and other immune weakening medications
4. People with severe obesity (body mass index [BMI] of 40 or higher)
5. People with diabetes
6. People with chronic kidney disease undergoing dialysis
7. People with liver disease

7. **Facilities Cleaning and Disinfection:** Summer camps have strong infection protocols in place and these protocols should be updated with EHE/CDC summer camp guidance when available to include:
   a. Clean and disinfect frequently touched surfaces at least daily (for example playground equipment, door handles, sink handles, etc.) and shared objects (for example, toys, games, art supplies) between uses.
   b. Clean and disinfect vans and buses; refer to guidance for bus transit operators. Camp vehicles should be cleaned and sanitized/disinfected between uses.
   c. Ensure safe and correct application of disinfectants per the manufacturers' instructions for use (IFU) by trained staff. Keep products away from children.
   d. Ensure ventilation systems operate properly and increase circulation of outdoor air as much as possible by opening screened windows and doors, using fans, or other methods. Do not open windows and doors if they pose a safety or health risk (e.g., temperature, inclement weather, insects, and allowing pollens in or exacerbating asthma symptoms) to children at the facility.
   e. Take steps to ensure that all potable water systems and terminal fixtures (for example, sinks and bottle filling stations) are sanitized daily. Provide disposable cups for water fountains and refillable water jugs. Avoid use of water bubblers without disposable cups.
   f. Ensure potable and process water plumbing systems are appropriately readied prior to camp in accord with CDC and local health department guidance to minimize the risk of diseases associated with waterborne pathogens.
8. **Transportation:** Camp directors are encouraged to arrange for camper and staff travel that minimizes exposures outside the camp community. This could include: charter buses, direct-to-camp/direct-to-home transportation. These guidelines are based upon the assumption that Camps are conducting pre-arrival screening prior to boarding buses to overnight camp. Air travel to camps from campers or staff are not permitted.

a. Create social distance between children on transport vans and buses where possible. Use face masks, if unable to maintain social distancing.
b. Camp administrators should be aware of the infection potential of campers and staff traveling from high infection transmission areas and are advised to consider limits to participants from these areas and/or in accord with the State and local agency requirements for regional, interstate, and international travel. If allowed, staff from these areas must quarantine in small groups (<10) for at least 14-days prior to arrival of campers or before introduction to camp and participate in pre-arrival screening.
c. Stagger arrival and drop-off/departure times or locations or put in place other protocols to limit direct contact with parents as much as possible.
d. Implement a one-parent, one-child drop off and pick up procedure. Parents should be ready to separate from child immediately after check-in and passed medical screening. Parents will not be allowed to enter housing areas prior to or during drop off.

9. **Communication with State and Local Public Health Authorities:** Residential camps will ensure timely and accurate reporting to the NH public health authorities for all notifiable diseases and conditions, including COVID. Camp directors should ensure a single point of contact for communication and familiarize themselves with NH public health reporting protocols and contact methods.

Business Process Adaptations

1. Please see extensive adaptation above in addition to applicable NH guidance for youth camp operation.
2. Some camps or programs may need to shorten or otherwise alter camp sessions or total operating season in order to accommodate this guidance.

SAFER AT HOME
OVERNIGHT CAMPS

CDC Guidance:

YOUTH PROGRAMS AND CAMPS DURING THE COVID-19 PANDEMIC

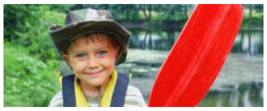

The purpose of this tool is to assist directors or administrators in making (re)opening decisions regarding youth programs and camps during the COVID-19 pandemic. It is important to check with state and local health officials and other partners to determine the most appropriate actions while adjusting to meet the unique needs and circumstances of the local community.

Should you consider opening?

- ✓ Will reopening be consistent with applicable state and local orders?
- ✓ Are you ready to protect children and employees at higher risk for severe illness?
- ✓ Are you able to screen children and employees upon arrival for symptoms and history of exposure?

ANY NO → **DO NOT OPEN**

ALL YES →

Are recommended health and safety actions in place?

- ✓ Promote healthy hygiene practices such as hand washing and employees wearing a cloth face covering, as feasible
- ✓ Intensify cleaning, disinfection, and ventilation of facilities and transport vehicles/buses
- ✓ Encourage social distancing through increased spacing, small groups, and limited mixing between groups, and staggered scheduling, arrival, and drop off, if feasible
- ✓ Where feasible, adjust activities and procedures to limit sharing of items such as toys, belongings, supplies, and equipment
- ✓ Train all employees on health and safety protocols

ANY NO ↓

ALL YES →

Is ongoing monitoring in place?

- ✓ Develop and implement procedures to check for signs and symptoms in children and employees daily upon arrival, as feasible
- ✓ If feasible, implement enhanced screening for children and employees who have recently been present in areas of high transmission, including temperature checks and symptom monitoring
- ✓ Encourage anyone who is sick to stay home
- ✓ Plan for if children or employees get sick
- ✓ Regularly communicate and monitor developments with local authorities, employees, and families regarding cases, exposures, and updates to policies and procedures
- ✓ Monitor child and employee absences and have a pool of trained substitutes, and flexible leave policies and practices
- ✓ Be ready to consult with the local health authorities if there are cases in the facility or an increase in cases in the local area

ANY NO ↓

ALL YES → **OPEN AND MONITOR**

cdc.gov/coronavirus

UPDATED JUNE 15, 2020
